# Protective Role of Endogenous Kallistatin in Vascular Injury and Senescence by Inhibiting Oxidative Stress and Inflammation

**DOI:** 10.1155/2018/4138560

**Published:** 2018-12-02

**Authors:** Julie Chao, Youming Guo, Lee Chao

**Affiliations:** Department of Biochemistry and Molecular Biology, Medical University of South Carolina, Charleston, SC, USA

## Abstract

Kallistatin was identified in human plasma as a tissue kallikrein-binding protein and a serine proteinase inhibitor. Kallistatin exerts pleiotropic effects on angiogenesis, oxidative stress, inflammation, apoptosis, fibrosis, and tumor growth. Kallistatin levels are markedly reduced in patients with coronary artery disease, sepsis, diabetic retinopathy, inflammatory bowel disease, pneumonia, and cancer. Moreover, plasma kallistatin levels are positively associated with leukocyte telomere length in young African Americans, indicating the involvement of kallistatin in aging. In addition, kallistatin treatment promotes vascular repair by increasing the migration and function of endothelial progenitor cells (EPCs). Kallistatin via its heparin-binding site antagonizes TNF-*α*-induced senescence and superoxide formation, while kallistatin's active site is essential for inhibiting miR-34a synthesis, thus elevating sirtuin 1 (SIRT1)/eNOS synthesis in EPCs. Kallistatin inhibits oxidative stress-induced cellular senescence by upregulating Let-7g synthesis, leading to modulate Let-7g-mediated miR-34a-SIRT1-eNOS signaling pathway in human endothelial cells. Exogenous kallistatin administration attenuates vascular injury and senescence in association with increased SIRT1 and eNOS levels and reduced miR-34a synthesis and NADPH oxidase activity, as well as TNF-*α* and ICAM-1 expression in the aortas of streptozotocin- (STZ-) induced diabetic mice. Conversely, endothelial-specific depletion of kallistatin aggravates vascular senescence, oxidative stress, and inflammation, with further reduction of Let-7g, SIRT1, and eNOS and elevation of miR-34a in mouse lung endothelial cells. Furthermore, systemic depletion of kallistatin exacerbates aortic injury, senescence, NADPH oxidase activity, and inflammatory gene expression in STZ-induced diabetic mice. These findings indicate that endogenous kallistatin displays a novel role in protection against vascular injury and senescence by inhibiting oxidative stress and inflammation.

## 1. Introduction

Aging is a major risk factor for the development of many diseases, including cardiovascular disease, stroke, and cancer [1–3]. Endothelial aging is associated with increased oxidative stress and inflammation and decreased endothelial nitric oxide synthase (eNOS) activity and nitric oxide (NO) production [4, 5]. Excessive oxidative stress and chronic inflammation are common causes of endothelial dysfunction in vascular disease and aging [4]. Oxidative stress impairs the mobility and function of endothelial progenitor cells (EPCs) and enhances cellular senescence [6]. EPCs play an integral role in vascular repair by the replenishment of damaged or senescent endothelial cells [7]. Circulating EPC number and function are markedly reduced in the aging population [8–11]. Kallistatin in human plasma has been identified as a tissue kallikrein inhibitor and a unique serine proteinase inhibitor [12–16]. Kallistatin exerts multifactorial activities, including vasodilation and inhibition of oxidative stress, inflammation, fibrosis, and apoptosis, primarily by increasing eNOS levels and NO formation [17–22]. Moreover, kallistatin administration increases circulating EPC number and reduces aortic oxidative stress, whereas kallistatin depletion augments endothelial cell loss, diminishes circulating EPC levels, and exacerbates renal and cardiovascular oxidative stress, inflammation, and organ remodeling in hypertensive rats [23, 24]. Kallistatin protein treatment enhances the migration and function of cultured human EPCs [23]. Furthermore, plasma kallistatin levels are positively associated with leukocyte telomere length in young African Americans [25]. Telomere length is critically related to vascular cell senescence, and NO prevents telomere shortening by stimulating telomerase activity [26, 27]. Therefore, kallistatin via NO formation may protect against vascular injury, senescence, and aging through its antioxidant and anti-inflammatory actions.

## 2. Reduced Kallistatin Levels in Vascular Disease and Metabolic Disorders

Kallistatin is mainly synthesized in the liver and distributed in blood vessels and organs relevant to cardiovascular function [14, 28–30]. Human kallistatin gene transcripts and protein can be detected in the plasma, liver, heart, lung, kidney, prostate gland, and aorta [14, 28, 29]. Kallistatin is localized in endothelial and smooth muscle cells of large, medium, and small blood vessels [30]. Kallistatin levels are reduced in a variety of human diseases, such as coronary artery disease, sepsis, severe pneumonia, and active pulmonary tuberculosis, as well as colon, prostate, and liver cancer [29, 31–35]. Moreover, reduced plasma kallistatin levels are associated with elevated obesity and cardiometabolic risk in apparently healthy African Americans [36]. Likewise, kallistatin levels are lower in vitreous fluids from patients with diabetic retinopathy and in the retinas of streptozotocin- (STZ-) induced diabetic rats [37, 38]. However, elevated serum kallistatin has been observed in patients with diabetic vascular complications [39]. Moreover, plasma kallistatin levels are markedly reduced in animal models of hypertension, STZ-induced diabetes mellitus, lipopolysaccharide- (LPS-) induced endotoxemia, renal injury, and hepatocellular carcinoma [15, 18, 29, 40–42]. Circulating kallistatin levels are negatively associated with elevated thiobarbituric acid reactive substance (TBARS, an indicator of oxidative stress) in diabetic rats [43]. In addition, kallistatin levels are reduced in animal models and humans with inflammatory disorders [29, 33, 44]. These combined findings indicate that kallistatin levels are reduced under excess oxidative stress and inflammation, implicating the involvement of kallistatin in vascular damage and metabolic disorders.

## 3. Kallistatin as an Effective Antioxidant and Anti-Inflammatory Agent

Oxidative stress is the main cause of endothelial injury and vascular disease states, and inflammation is known to increase with aging [45, 46]. Kallistatin administration attenuates hypertension and organ damage in conjunction with increased eNOS and NO levels and reduced oxidative stress and inflammation in animal models [18, 19, 22, 23]. Kallistatin administration markedly reduces inflammatory responses in animal models of arthritis, hypertension, myocardial ischemia, and septic shock [17, 19, 21, 22, 47, 48]. Moreover, kallistatin overexpression protects against diabetic retinopathy in *db*/*db* mice by multiple mechanisms, including antioxidant, anti-inflammatory, antifibrotic, and blood pressure-lowering effects [49]. NO has antioxidant properties by inhibiting NADPH oxidase activity [50]. Kallistatin, via NO formation, reduces H_2_O_2_- or angiotensin II-induced NADPH oxidase activity and reactive oxygen species (ROS) formation in cultured endothelial cells, renal tubular cells, and cardiomyocytes [18, 22, 47]. Kallistatin inhibits vascular inflammation and apoptosis by stimulating eNOS synthesis and activation and NO formation [22, 51], as well as by preventing TNF-*α*- and high-mobility group box protein 1- (HMGB1-) mediated inflammatory gene expression [17, 21]. Thus, kallistatin appears to be a unique antioxidant and anti-inflammatory agent.

## 4. Kallistatin Reduces Senescence in EPCs and Endothelial Cells

EPCs are a subset of mononuclear cells derived from the bone marrow that have the ability to differentiate into mature endothelial cells [52]. Reduced EPC number is associated with defective proliferation and mobility as well as accelerated apoptosis and senescence [53]. Kallistatin depletion by neutralizing antibody injection augments glomerular endothelial cell loss and diminishes circulating EPC numbers [23, 24]. Excess oxidative stress or inflammation impairs the mobility and function of EPCs and increases cellular senescence [54, 55]. Indeed, recombinant human kallistatin treatment significantly inhibits TNF-*α*-induced EPC senescence by blocking oxidative stress and reducing the senescent markers plasminogen activator inhibitor (PAI)-1, microRNA- (miR-) 21, and p16^INK4a^, while increasing telomerase activity [56]. The miR-34a-sirtuin 1 (SIRT1) axis is a key pathway in the aging process [57]. Kallistatin via its active site inhibits prosenescent miR-34a expression and antagonizes miR-34a-mediated inhibition of the antioxidant enzymes SIRT1 and eNOS in EPCs [56]. As a deacetylase, SIRT1 stimulates antioxidant enzymes including eNOS, catalase, and superoxide dismutase (SOD), and eNOS through NO production stimulates SIRT1 enzymatic activity and inhibits NADPH oxidase activity [58, 59], leading to the attenuation of oxidative stress. Similarly, kallistatin exerts salutary effects in the setting of H_2_O_2_-induced endothelial senescence, oxidative stress, and inflammation, as indicated by reduced p16^INK4a^, PAI-1, and miR-34a synthesis, NADPH oxidase activity/expression, vascular cell adhesion molecule- (VCAM-) 1 and intercellular adhesion molecule- (ICAM-) 1 expression, and increased telomerase activity in endothelial cells [60]. Moreover, kallistatin via upregulating the endoprotective miRNA Let-7g coordinates Let-7g-modulated miR-34a-SIRT1-eNOS pathway and achieves antisenescent, antioxidant, and anti-inflammatory actions in endothelial cells [60]. Activation of SIRT1-eNOS signaling results in increased catalase and NO levels, thus suppressing oxidative vascular damage [60]. These combined studies reveal the mechanisms of kallistatin in protection against vascular injury by reducing cellular senescence in EPCs and endothelial cells.

## 5. Kallistatin Treatment Attenuates Vascular Injury and Senescence in Animal Models

**Figure 1:**
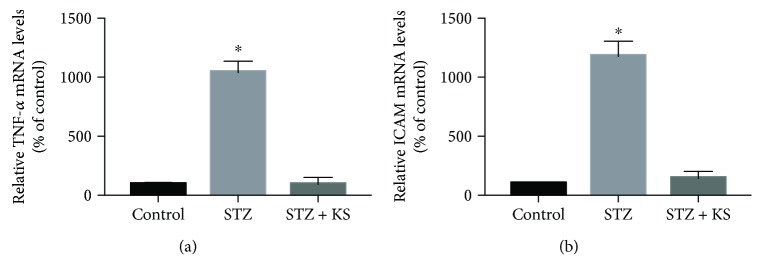
Kallistatin treatment on TNF-*α* and ICAM-1 synthesis in the aorta of STZ-induced diabetic mice. Values are expressed as mean ± SEM. *n* = 3. ^∗^*P* < 0.05 vs. the control group.

As age advances, the alteration of vascular structure and function is intensified, such as endothelial senescence/rarefaction and increased oxidative stress and inflammation [61]. High blood glucose-induced vascular complications are manifested in diabetes [62]. The STZ-induced diabetic mouse is a popular model for studying vascular injury and aging [63, 64]. Kallistatin protein treatment inhibits aortic senescence and superoxide formation in association with reduced miR-34a synthesis and increased SIRT1 and eNOS expression in STZ-induced diabetic mice [56]. Moreover, kallistatin administration significantly inhibits inflammatory gene expression, such as TNF-*α* and ICAM-1, in the aorta of STZ-induced diabetic mice (Figure 1), indicating the beneficial effect of kallistatin in diabetes-associated vascular senescence, oxidative stress, and inflammation. In addition to diabetes, kallistatin gene delivery suppresses aortic superoxide formation and glomerular capillary loss in salt-induced hypertensive rats [22]. Likewise, kallistatin treatment attenuates cardiac dysfunction, apoptosis, and inflammation in conjunction with decreased oxidative stress and increased eNOS and NO levels in animal models of hypertension and myocardial infarction and ischemia-reperfusion injury [18, 19, 22, 47, 65]. Furthermore, kallistatin gene delivery inhibits vascular leakage in mice provoked by C5a and prevents arthritis-induced inflammation in rats [21]. Importantly, kallistatin is capable of improving oxidative stress-induced survival/aging at the organismal level in *Caenorhabditis elegans* by regulating the miR-34a-SIRT1 pathway [56]. Thus, as a potent antioxidant and anti-inflammatory agent, kallistatin could have a significant impact on vascular injury and senescence.

## 6. Endothelial-Specific Depletion of Kallistatin Exacerbates Cellular Senescence, Oxidative Stress, and Inflammation in Mouse Endothelial Cells

Endothelial-specific kallistatin knockout (KS^endo−/−^) mice were generated by Cre-loxp recombination for exploring the role of endogenous kallistatin in diabetes-associated endothelial senescence, oxidative stress, and inflammation [60]. Mouse lung endothelial cells were isolated from 8-week-old KS^endo−/−^ and wild-type (WT) mice with CD31 immunoselection and cultured as described [66]. Kallistatin deficiency in mouse lung endothelial cells displayed aggravated senescence, oxidative stress, and inflammation, as evidenced by increases in senescence-associated (SA) *β*-gal activity, PAI-1/p16^INK4a^ synthesis, superoxide formation, and expression of the inflammatory genes ICAM-1, VCAM-1, and interleukin- (IL-) 6 [60]. Kallistatin deficiency in endothelial cells exacerbated oxidative stress-induced senescence, superoxide formation, NADPH oxidase activity, and inflammatory gene expression in endothelial cells, indicating a protective role of endogenous kallistatin in maintaining endothelial viability and function. Moreover, endothelial-specific depletion of kallistatin elevated senescence inducer miR-34a but reduced Let-7g and antioxidant genes, including SIRT1, eNOS, and catalase [60]. These findings support the notion that endogenous kallistatin acts as a protective molecule in endothelial senescence by inhibiting oxidative stress and inflammation.

## 7. Systemic Depletion of Kallistatin Aggregates Vascular Injury, Senescence, Oxidative Stress, and Inflammation in Diabetic Mice

**Figure 2:**
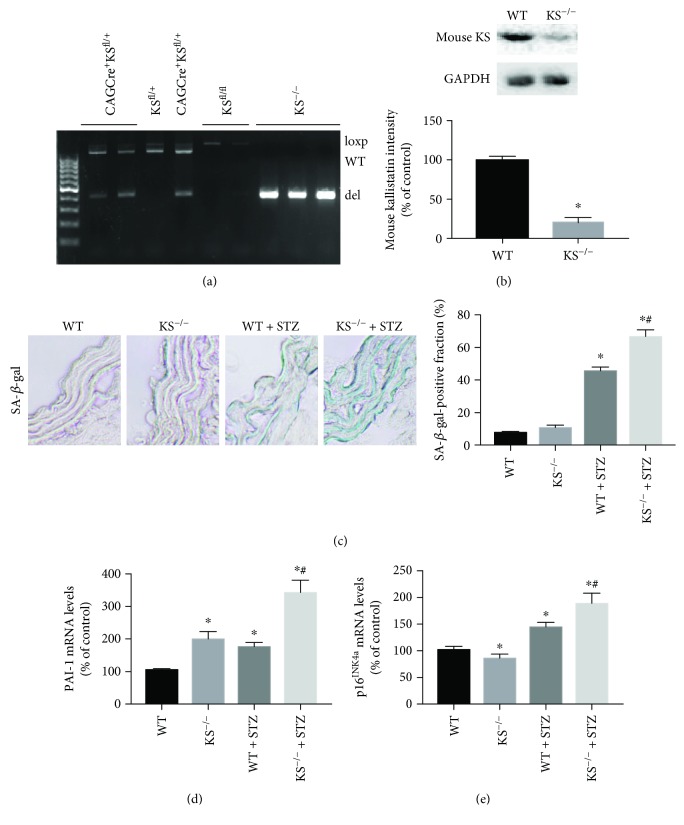
Systemic depletion of kallistatin exacerbates aortic senescence. (a) Kallistatin depletion identified by genotyping with the expression of loxp, wild-type (WT) and deletion (del) alleles. (b) Western blot analysis of mouse kallistatin expression in the kidney of WT mice and KS^−/−^ mice. (c) Representative images and quantitative analysis of *β*-gal staining of aorta sections from WT, KS^−/−^ mice with or without STZ treatment. (d, e) PAI-1 and P16^INK4a^ mRNA levels in mouse aorta. Values are expressed as mean ± SEM. *n* = 3. ^∗^*P* < 0.05 vs. the WT group, ^#^*P* < 0.05 vs. the WT + STZ group.

**Figure 3:**
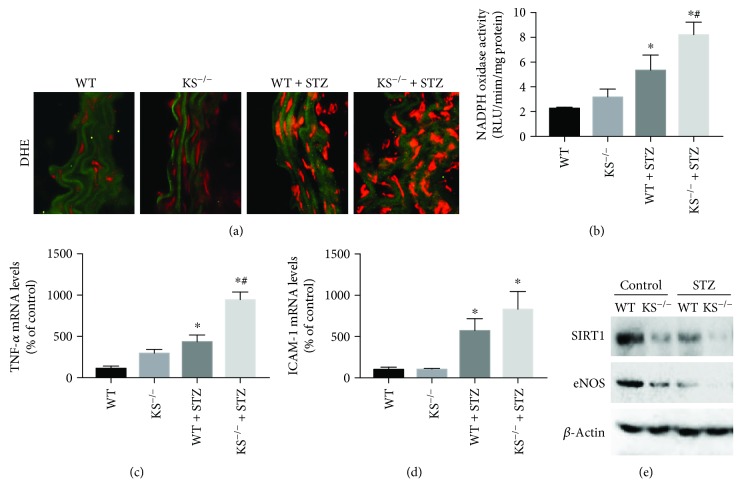
Systemic depletion of kallistatin exacerbates aortic oxidative stress and inflammation. (a) Representative images of superoxide formation in aortas from WT, KS^−/−^ mice with or without STZ treatment, as indicated by red fluorescence dye DHE. Collagen fibers displayed green autofluorescence in the aorta. (b) NADPH oxidase in mouse aorta. (c) Representative western blot of SIRT1 and eNOS expression in mouse aorta. (d&e) Inflammatory gene expression of TNF-*α* and ICAM-1 in mouse aorta. Values are expressed as mean ± SEM. *n* = 3. ^∗^*P* < 0.05 vs. WT group, ^#^*P* < 0.05 vs. WT + STZ group.

Kallistatin depletion by neutralizing antibody injection aggravates aortic oxidative stress, capillary loss, and cardiovascular and renal remodeling, accompanied by elevated oxidative stress and inflammation in hypertensive rats [23, 24]. To further investigate the role of endogenous kallistatin in vascular injury and senescence, we generated systemic deficiency of kallistatin (KS^−/−^) mice by Cre-loxp recombination technology [60]. Briefly, female homozygous floxed KS^fl/fl^ mice were crossed with male CAGCre^+^ mice for two generations to obtain CAGCre^+^KS^fl/fl^ mice. Tamoxifen was injected in these mice to induce Cre recombinase activity. The genotyping result showed that only KS^−/−^ mice were positive for deletion allele expression and negative for loxp and WT allele expression (Figure 2(a)). Kallistatin depletion was further identified by reduced mouse kallistatin protein levels in western blot analysis in the kidney of KS^−/−^ mice, compared with WT mice (Figure 2(b)). In contrast with kallistatin's efficacy in aortic senescence and oxidative stress in STZ-induced diabetic mice [56], systemic depletion of kallistatin worsened vascular damage and senescence, characterized by aggravated aortic thickening, structural disarrangement, elevated SA-*β*-gal activity, and PAI-1 and p16^INK4a^ mRNA levels in the aorta of diabetic mice (Figures 2(c)–2(e)). Kallistatin depletion also worsened aortic collagen deposition, superoxide formation, and NADPH oxidase activity and elevated inflammatory gene expression, including TNF-*α* and ICAM-1 (Figures 3(a)–3(d)). Moreover, levels of the antioxidant proteins SIRT1 and eNOS were significantly reduced in the aorta of KS^−/−^ mice with STZ-induced diabetes (Figure 3(e)), in contrast to the stimulatory effect of kallistatin administration on SIRT1 and eNOS expression [56]. These combined findings indicate that endogenous kallistatin is a protective agent against vascular injury and senescence.

## 8. Conclusion

**Figure 4:**
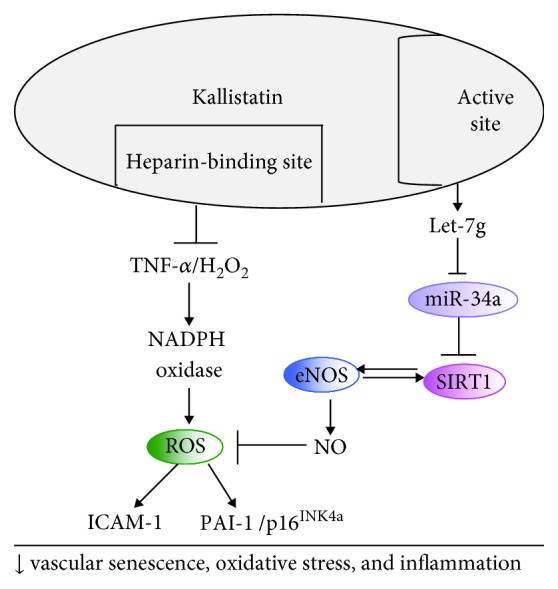
Signaling pathways of kallistatin in protection against vascular senescence, oxidative stress, and inflammation in EPCs and endothelial cells. Kallistatin via its heparin-binding site blocks TNF-*α*/H_2_O_2_-modulated NADPH oxidase activity and ROS formation and thus ICAM-1 and PAI-1/p16^INK4a^ expression. Kallistatin through its active site stimulates Let-7g synthesis, to inhibit miR-34 and stimulate SIRT1-eNOS pathway, thereby elevating NO levels, further leading to reduced ROS levels.

These combined studies reveal a novel role of endogenous kallistatin in protection against vascular injury and senescence. Kallistatin reduces vascular injury and senescence by promoting the migration and function of EPCs. Exogenous kallistatin treatment attenuates aortic injury, senescence, oxidative stress, and inflammation in animal models, while kallistatin deficiency in endothelial-specific or general knockout mice exacerbates vascular injury, senescence, oxidative stress, and inflammation in primarily cultured mouse endothelial cells and diabetic mice. The signaling pathways by which kallistatin inhibits vascular senescence, oxidative stress, and inflammation in EPCs and endothelial cells are shown in Figure 4. Kallistatin is an endogenous protein with no cytotoxic effects. Therefore, kallistatin could potentially be used as an effective therapeutic regimen for aging-associated vascular disease in humans.
